# Safe inhalation pipe provision (SIPP): protocol for a mixed-method evaluation of an intervention to improve health outcomes and service engagement among people who use crack cocaine in England

**DOI:** 10.1186/s12954-024-00938-7

**Published:** 2024-01-23

**Authors:** Magdalena Harris, Jenny Scott, Vivian Hope, Joanna Busza, Sedona Sweeney, Andrew Preston, Mat Southwell, Niamh Eastwood, Cedomir Vuckovic, Caitlynne McGaff, Ian Yoon, Louise Wilkins, Shoba Ram, Catherine Lord, Philippe Bonnet, Peter Furlong, Natasha Simpson, Holly Slater, Lucy Platt

**Affiliations:** 1 Department of Public Health, Environments and Society, London School of Hygiene and Tropical Medicine, 15-17 Tavistock Place, London, WC1H 9SH UK; 2 Centre for Academic Primary Care, Bristol Medical School, University of Bristol, Canynge Hall, Bristol, BS8 2PS UK; 3 Public Health Institute/School of Public and Allied Health, Liverpool John Moores University, 3rd Floor Exchange Station, Tithebarn Street, Liverpool, L2 2QP UK; 4 Department of Global Health and Development, LSHTM, 15-17 Tavistock Place, London, WC1H 9SH UK; 5 Exchange Supplies, 1 Great Western Industrial Centre, Dorchester, Dorset DT1 1 UK; 6 Coact, 2 Crescent Place Mews, Bath, BA2 2PY UK; 7 Release, 61 Mansell Street, London, E1 8AN UK; 8 The Health Shop, 12 Broad Street, Nottingham, NG1 3AL UK; 9 The Maples, Verona House, 53 Filwood Rd, Bristol, BS16 3RX UK; 10 Bristol Drugs Project, 11 Brunswick Square, St Paul’s, Bristol, BS2 8PE UK; 11 The Hepatitis C Trust, 72 Weston Street, London, SE1 3QG UK; 12 Change Grow Live, 34 Albion Place, Leeds, LS1 6JH UK; 13 Cranstoun Sandwell, 128B Oldbury Rd, Smethwick, B66 1JE UK; 14 POW Nottingham, 16 Independent Street, Nottingham, NG7 3LN UK

**Keywords:** Crack cocaine, Crack pipe, Harm reduction, Intervention, Evaluation, Participatory research, Health outcomes, Service engagement

## Abstract

**Background:**

Over 180,000 people use crack cocaine in England, yet provision of smoking equipment to support safer crack use is prohibited under UK law. Pipes used for crack cocaine smoking are often homemade and/or in short supply, leading to pipe sharing and injuries from use of unsafe materials. This increases risk of viral infection and respiratory harm among a marginalised underserved population. International evaluations suggest crack pipe supply leads to sustained reductions in pipe sharing and use of homemade equipment; increased health risk awareness; improved service access; reduction in injecting and crack-related health problems. In this paper, we introduce the protocol for the NIHR-funded SIPP (Safe inhalation pipe provision) project and discuss implications for impact.

**Methods:**

The SIPP study will develop, implement and evaluate a crack smoking equipment and training intervention to be distributed through peer networks and specialist drug services in England. Study components comprise: (1) peer-network capacity building and co-production; (2) a pre- and post-intervention survey at intervention and non-equivalent control sites; (3) a mixed-method process evaluation; and (4) an economic evaluation. Participant eligibility criteria are use of crack within the past 28 days, with a survey sample of ~ 740 for each impact evaluation survey point and ~ 40 for qualitative process evaluation interviews. Our primary outcome measure is pipe sharing within the past 28 days, with secondary outcomes pertaining to use of homemade pipes, service engagement, injecting practice and acute health harms.

**Anticipated impact:**

SIPP aims to reduce crack use risk practices and associated health harms; including through increasing crack harm reduction awareness among service providers and peers. Implementation has only been possible with local police approvals. Our goal is to generate an evidence base to inform review of the legislation prohibiting crack pipe supply in the UK. This holds potential to transform harm reduction service provision and engagement nationally.

**Conclusion:**

People who smoke crack cocaine in England currently have little reason to engage with harm reduction and drug services. Little is known about this growing population. This study will provide insight into population characteristics, unmet need and the case for legislative reform.

*Trial registration***:** ISRCTN12541454 https://doi.org/10.1186/ISRCTN12541454

## Background

The use of crack cocaine, either via inhalation or injection, is associated with adverse health and social outcomes, such as infectious disease, respiratory harms, premature mortality, acquisitive crime engagement and incarceration [1–6]. England has the highest prevalence of crack use in the European Region [7]. The population of people who use crack (PWUC) is growing, with an 8.5% rise from an estimated 166,640 in 2012 to 180,748 in 2017 [8]. An official inquiry into this increase highlights it as a serious public health concern, calling for “research to explore the characteristics of ‘hidden’ crack users who are not currently in treatment” [5]. In England, most of what is known about crack use is drawn from drug treatment and criminal justice services, and thus mostly relates to people who also inject heroin, access services for opioid substitution therapy (OST) and/or are in contact with the criminal justice system [9]. Modelling data from 2017 estimated that 29% of people who use crack in England (52,677) do not use heroin or OST and are thus less likely to be in touch with specialist drug services [8].

Provision of OST and needle and syringe programmes (NSP) are evidenced lifesaving interventions that directly benefit those receiving them by reducing risk, such as hepatitis C virus (HCV) and HIV transmission [10], but also provide indirect benefits, through facilitating links to health and social care services. While OST offers protection against heroin withdrawal, there is no commensurate pharmaceutical treatment available for crack cocaine use or dependency. Psychological treatment for cocaine dependence is advocated [11], yet motivation to engage with services can be low [5, 12]. This is particularly the case for people who smoke crack cocaine, given that in the UK provision of equipment to facilitate safer crack smoking practices is prohibited under the Sect. 9A of the Misuse of Drugs Act, 1971 [13].

There is a growing body of evidence showing associations between crack smoking and HIV and HCV acquisition, attributed to sharing of pipes [1, 2, 14–17]. Provision of injecting equipment in isolation from pipe provision, may support the choice of injection as the mode of crack administration [18]. Crack injection, often in combination with heroin, is common in England and Wales, with 54% of people who inject drugs reporting recent crack injection in 2020–21 [19]. Crack injection is associated with elevated blood-borne virus (HCV, HIV) and bacterial infection risk, given increased injection frequency compared to opioid use [20, 21]. In England, pipes used for smoking crack are often homemade or repurposed [12]. Depending on the materials employed, this can heighten risk of respiratory harm from fume and particulate inhalation with long term sequalae such as chronic obstructive pulmonary disease (COPD) as well as acute cuts and burns from using sharp or broken implements [22–26]. This in turn can increase blood-borne virus transmission risk [16].

International evidence indicates that crack pipe supply through harm reduction and drug treatment services can reduce injecting, acute injuries, respiratory harms and viral infections, and engage people with drug treatment services where health, harm reduction, and psychological interventions can be delivered [1, 2, 18, 27–32]. The quality of this body of work is limited, with a small number of published studies, of methodological heterogeneity and primarily composing observational and uncontrolled designs. Most of this work originates from Canada, where crack pipe provision is legal, and contextual differences mean that findings might not be transferable. Given prohibitions on crack pipe supply, there is a dearth of evidence on associated interventions in the UK context, although reports from Ireland, where crack pipe provision is legal, demonstrate strong intervention engagement and acceptability among highly marginalised and at-risk populations [33].

In this paper, we introduce the National Institute for Health and Care Research (NIHR)-funded SIPP (Safe inhalation pipe provision) study, which commenced in July 2022 and will run until February 2025. We provide the study protocol and discuss implications for impact. To our knowledge, this study is the first of its kind globally and has been designed to meet a pressing need in the UK context, given increasing use of crack cocaine and the high prevalence of COPD among people who smoke crack and/or heroin [23, 34–36]. Alert to the heightened risk of pipe sharing and unsafe equipment use for people with respiratory vulnerabilities, members of the study team advocated for a relaxation of restrictions to allow for crack pipe provision during the COVID-19 pandemic. This application, for a government supported memorandum of understanding between police and providers, was unsuccessful due to concerns about promotion of crack use and a dearth of UK evidence [12]. The SIPP study was designed to address these concerns, with a methodologically rigorous pre- and post-intervention survey component with non-equivalent control sites, complemented by a process evaluation and peer research capacity building in geographically diverse sites, as outlined below. Our study is designed to answer the question: “To what extent and how does SIPP reduce health risks and enhance service engagement among people who use crack cocaine?”.

### The SIPP study

**Fig. 1:**
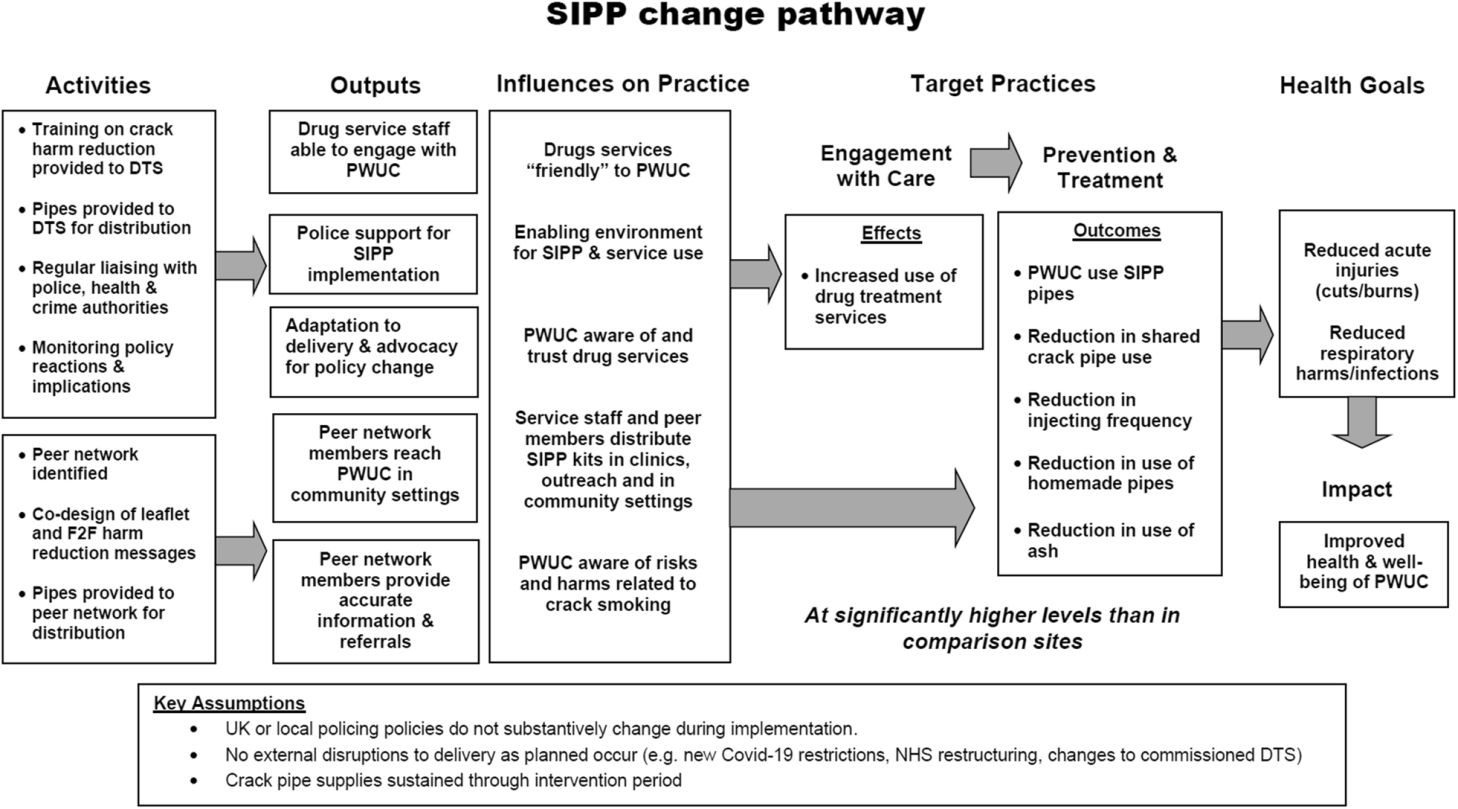
SIPP logic model

The SIPP study is a mixed-method evaluation of an intervention to distribute crack equipment and harm reduction information, through specialist services for people who use drugs (e.g. NSPs, sex worker services, and other harm reduction interventions) and peer networks in England. The aim of the intervention is to reduce crack smoking risk practices and associated health harms, including through increasing harm reduction knowledge and confidence among drug treatment service providers and peers. Our goal is to reduce health and service access inequity among people who use crack, by building an evidence base to inform service provision and promote review of the legislation prohibiting crack pipe supply. Our proposed pathway to achieve change is outlined below (Fig. 1).

We employ a mixed-methods quasi-experimental design to examine whether, to what extent and how SIPP acts to reduce health risks and enhance service engagement among people who use crack cocaine. Objectives, detailed in Table 1, will be achieved through the implementation of: (1) peer-network capacity building and co-production; (2) an impact evaluation comprising a pre- and post-intervention survey with a non-equivalent control group; (3) a process evaluation comprising ethnographic observations, qualitative interviews and monitoring data; (4) an economic evaluation. Detail on each study component is provided below, after a brief outline of the SIPP intervention and study sites.

**Table 1:** SIPP study objectives and components

SIPP objective	Study component
1. Build peer-network research capacity and explore whether the quality and impact of their SIPP engagement with PWUC differs in comparison with SIPP engagement through drug treatment services	Peer network
	Process evaluation; Impact evaluation
2. Measure the effect and cost-effectiveness of the SIPP kit on harms and risks associated with crack use (pipe sharing, presentation at drug services, using homemade pipes, cuts/burns, crack injecting)	Impact evaluation; economic evaluation
3. Evaluate SIPP fidelity, reach and acceptability in diverse specialist drug service and peer-network settings	Process evaluation
4. Explore the barriers and facilitators to SIPP uptake and service engagement among PWUC	Process evaluation
5. Explore the mechanisms through which SIPP facilitates changes in health risks and access to services, and how these are shaped by local contextual factors	Process evaluation
6. Co-develop a scalable SIPP toolkit and harm reduction resources to enhance PWUC engagement with specialist drug services and to facilitate crack-related risk reduction practices	Peer network
7. Translate evidence to policy and advocacy outputs, including to inform legislative review	All components

### The SIPP intervention

SIPP is a structural intervention. That is, an intervention that promotes the availability, accessibility or acceptability of specific resources needed for specific health outcomes [37]. It is informed by comparable interventions, harm reduction principles and models of community mobilisation evidenced to have impact in diverse contexts [38, 39]. The SIPP intervention was co-produced by the team with input from people who use crack and service providers and will be delivered at study intervention sites for a period of six months.

**Fig. 2:**
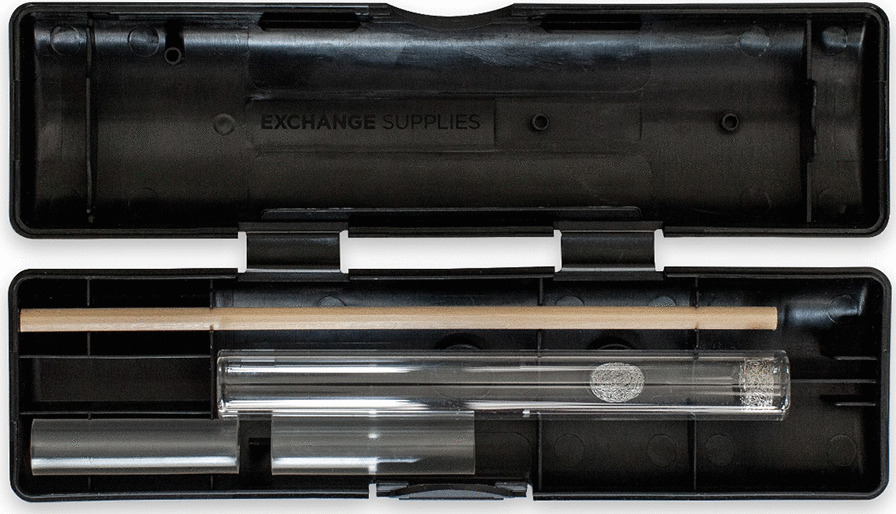
SIPP Kit

It consists of three components:

1. The SIPP KIT: The SIPP kit adheres to best practice guidance for provision of safe crack inhalation equipment [32]. It comprises a straight stem borosilicate glass pipe; 2 × steel gauze filters/meshes; 2 × plastic mouth pieces; and a wooden push stick, contained within a discrete hard plastic case. A brief harm reduction leaflet with instructions and a card providing information about the research study are provided with each kit (see Fig. 2)
2. SIPP Provider Training: Informed by an evidence review, workshops with providers of specialist services for people who use drugs, and peer/stakeholder review, we developed an online training module aimed at service providers. This provides background information on crack (drug properties, use in the UK context); associated health harms and harm reduction advice; homemade pipes, suspension devices (ash, wire wool) and risk; client engagement tips; SIPP kit use and provision. Training evaluation and participant registration details are embedded within the platform.
3. Peer-2-peer harm reduction: Brief verbal harm reduction messaging to support SIPP kit provision. With peers (i.e. people who use crack) we developed a hierarchy of harm reduction messages that can be tailored to suit an individual's specific information requirements and time available. The development and provision of this messaging is an iterative process, informed by feedback from peer researchers on local participant needs and contexts of delivery.

### Study partners and sites

The SIPP study would not be possible without the support of local police forces, specialist drug service providers, community members and charity organisations working to support peer advocacy and involvement. Prohibition of crack pipe provision necessitated contact with local Chief Constables and Police and Crime Commissioners prior to study site selection. Our intervention sites in Bristol, Nottingham, and Mansfield, are under the jurisdiction of the Avon & Somerset Police and the Nottinghamshire Police, respectively. Both have provided written approval for SIPP kit distribution through our specialist drug service providers and peer-network partners for the six-month duration of the intervention.

Non-equivalent control sites have been selected for intervention site comparability, with attention to local geography, demographics and the number of people using specialist services for drug use type recorded as using crack cocaine. Geographical distance between control and intervention sites decreases contamination risk. The control sites are: Birmingham (for Bristol, conurbations); Coventry (for Nottingham, mid-sized cities); Leamington Spa and Nuneaton in Warwickshire (for Mansfield, Nottinghamshire, peri-rural/rural sites adjoining Coventry and Nottingham, respectively). Each intervention and control site has a primary partner who is specialist provider of services for people who use drugs, with additional peer network and outreach distribution supported by Coact (Bristol), The Hepatitis C Trust (Birmingham) and POW sex worker support service and outreach (Nottingham). See Table 2, below.

**Table 2:** Intervention and non-equivalent control sites

Service type	Intervention site	Non-equivalent control site
Specialist drug service	Nottingham Health Shop/Well-being Hub	Change Grow Live—Coventry
Sex worker support service	POW Nottingham	
Specialist drug service	Bristol Drugs Project	Change Grow Live—Birmingham west
Peer network	Bristol COACT peer activist group	Hepatitis C Trust peer outreach—Birmingham
Specialist drug service	Change Grow Live –Nottinghamshire	Change Grow Live—Warwickshire

### Study population

Our study population for the evaluation are people who use crack cocaine, defined as people who self-report smoking or injecting crack in the past 28 days. To participate they must be aged 18 years or over and have the capacity to consent. Excluded are those aged under 18 years; currently incarcerated; lacking capacity for informed consent; or with no history of crack use. We include people who inject crack as a SIPP target population, given the potential for inhalation equipment provision to support transitions from injection and reduce injection frequency.

### Ethical issues

Ethical approval has been granted by The London School of Hygiene & Tropical Medicine (LSHTM) Research Ethics Committee (REF: 28102). Consultation has taken place with specialist drug service providers, their clients and organisations representing people who use drugs to ascertain that the project design does not place any undue burden or risk on potential participants. The co-production of the intervention with people who use crack and the embedded role of peers throughout, including as members of the research team and advisory board, helps to ensure that the project is acceptable and accountable to the communities involved.

Prior to project commencement the team will provide staff and volunteers at the specialist drug services training in research methods, with emphasis on prioritising the confidentiality and autonomy of people from marginalised and criminalised populations. All services have been provided with a list of local health and social support services to ensure a range of support options to participants where needed. All participants will be informed of what will happen to their data and measures taken to ensure confidentiality, prior to providing consent. De-identified qualitative interview transcripts will be encrypted and stored on password-protected LSHTM computers. Audio files will be destroyed once transcribed. All questionnaire data will be collected via the Open Data Kit software (ODK collect) on handheld password-protected tablet devices. Once each questionnaire is finalised, ODK Connect applies an asymmetric public key encryption using 256-bit encryption, which is irreversible and ensures that the finalised questionnaire data are not readable and are not tampered with. The encrypted form is sent to a central server hosted at LSHTM and downloaded to a secure server by the team data manager.

The safety of peers and providers engaged in the intervention delivery is paramount. Intervention police forces have provided written assurance that our specified peers and providers are exempt from prosecution for crack pipe supply. The team have met with and provided training to one police force, in particular, who have also nominated to provide peers with additional protection through the development of police approved identification badges including a study specific code which will inform concerned officers of the study remit and exemptions from prosecution. All peers involved in the project receive training in research methods and regular debriefs and check-ins with the qualitative research team. Throughout, it is stressed that peer safety and well-being take prominence over data generation and if a peer wishes to cease or change their role they will be supported to do so.

The ethics of intervention cessation are a concern, particularly if crack pipe provision is found to be highly valued and associated with positive outcomes for people who use crack. We have put in place measures to manage expectations of provision, including a card to be provided with each SIPP kit and posters to be displayed prominently in services. Both highlight that SIPP crack pipe provision is a time limited intervention, taking place as part of a research study. Although SIPP is not a clinical intervention, participant experiences in heroin-assisted therapy trials such as SALOME and NAOMI in Canada [40] demonstrate the need for caution. Here, the lack of an appropriate exit strategy meant that participants who experienced life-changing benefits from heroin-assisted therapy were required, at the end of the trial, to return to conventional treatments that had previously failed them. In response the SALOME/NAOMI Association of Patients filed a Supreme Court constitutional challenge to the trial discontinuation procedures, as part of their advocacy for treatment continuation [40]. Although not of the same magnitude, the potential for crack pipe provision to offer an avenue for meaningful service engagement and/or reduce risk practices and acute health harms, poses ethical concerns regarding intervention cessation. This is further complicated by the legal status of provision, with police approvals only in place for the intervention duration.

### Study components

#### 1. Peer capacity building and co-production

Community mobilisation and capacity building are central to the SIPP project design. We adopt a multi-pronged recruitment approach, working with community-based specialist services for people who use drugs but also peer activists and networks who are experienced in delivering harm reduction interventions to their communities. Through a dynamic process of peer researcher development and community mobilisation we will capacitate and evaluate peer-led provision of the SIPP intervention alongside provision through more traditional harm reduction services. Peers (i.e. people with past and/or present experience of crack use) in three sites (Bristol, Nottingham and Birmingham) will receive training in research methods, including those specific to SIPP, and be supported by the team to deliver each study component as appropriate to their location (intervention/control). At each site, 3–5 peers will generate pre- and post-survey data with a focus on recruiting people not accessing drug treatment services, women, and ethnic minorities. During the six-month intervention period peers in the intervention sites will distribute pipes and harm reduction advice, with a focus on reaching the underserved communities recruited during survey generation. All peer researchers will be remunerated for their time and expenses, including for training participation.

People who use crack have privileged access to the communities in which they live. Our peer-network team members provide unprecedented access to people who use crack who are not in touch with services. Peers are instrumental in transferring crack risk reduction knowledge [38] and providing support in the context of drug use, homelessness and exclusion [39, 41]. This project will add value by seeking to understand the barriers and facilitators to working with peer networks as research partners to inform future community-participatory research with marginalised populations. In addition, we will explore if the mode of SIPP delivery (peer network vs. drug treatment service) influences SIPP acceptability and reduction in risk practices. This knowledge has transferable value to inform scale up of other harm reduction and public health interventions for marginalised populations (NSP, vaccine rollout, etc.).

The team have extensive experience of working with community members including to build research capacity, and to develop peer-led harm reduction initiatives in contexts of service constraint and inaccessibility. The study principal investigator and two SIPP co-investigators have past & present experience of crack use. We have drawn on their connections to work closely with peers throughout the development of this proposal to arrive at an implementation and evaluation design that is feasible and acceptable to peer researchers, including in regard to modes of recruitment, data collection devices, training needs, reimbursements provided and control measures. In addition, the specialist services for people who use drugs in each site have been provided with funding to employ a service user or early career staff member with lived experience to administer the pre- and post-intervention survey. This reduces drug treatment staff burden and capacitates a local peer volunteer in research methods. Findings will be regularly fed back to and interpretation discussed with peer researchers and their networks, including through analysis, resource development and dissemination workshops.

#### 2. Impact evaluation

We will implement a pre-post-intervention survey at the intervention and control sites to measure the impact of SIPP on our primary and secondary outcomes. The survey will run for 10 weeks either side of the six-month intervention period and will be administered by trained staff and peers at each specialist service for people who use drugs, and through peer-network outreach in Bristol, Nottingham and Birmingham.

##### Data collection

Two surveys will be conducted six months apart to allow for the intervention phase. For both the pre- and post-survey, a structured questionnaire will be administered by staff at specialist drug services and peer researchers, trained in data collection methods, informed consent procedures and all aspects of the study protocol. Service providers and peers will approach potential participants and current clients, describe the study and discuss contents of the information sheet and consent form. Participants who consent will complete a structured questionnaire in English on a Lenovo tablet device with the ODK collect app either self-completed or administered by a service provider or peer. The questionnaire takes approximately 30 min to complete and contains the follow sections: demographics (including age, gender identity, sexual orientation, ethnicity, and source(s) of income); drug use practices (including drugs taken, routes of administration, and equipment sharing/reuse); indicators of social exclusion (including housing status and healthcare access); policing (including recent arrest and history of incarceration); primary and secondary outcomes (see below). Our questionnaire has been developed with input from peers and service providers and incorporates measures used in comparable surveys with people who use crack in Canada and UK [2, 18, 27, 28, 30, 42] as well as drawing on a questionnaire piloted by peers in 2020 for SIPP study conceptualisation. We will not actively follow-up participants, but we will collect minimal identifiers (date of birth, initials) needed to link people over time to allow assessment of primary and secondary outcomes and new presentations at specialist drug services. The same questionnaire will be used to collect post-intervention survey data.

##### Outcome measures

Our primary outcome measure is a decrease in the proportion of participants self-reporting sharing of crack pipes in the past 28 days (yes/no).

Secondary outcomes comprise:

1. increased presentations at specialist drug services defined as: a) the change in proportion of attendances at specialist drug service sites in the past 6 months (number of new attenders in past 6 months/total number of clients) measured through data linkage to drug service records; and b) the change in proportion of participants attending a specialist drug service in the past 6 months (measured through self-report yes/no)
2. reduction in injecting frequency defined as number of times injected in the past 28 days
3. reduction in number of days sharing pipes in the past 28 days
4. decrease in proportion of those reporting current acute injuries defined as cuts/burns to mouth or lips in the past 28 days (yes/no)
5. reduction in the proportion of participants who use homemade pipes in the past 28 days (yes/no)
6. reduction in proportion of participants using ash as a crack suspension device in the past 28 days (yes/no)
7. reduction in proportion of participants reporting respiratory risk markers defined as difficulty breathing, chest pain, coughing blood in the past 28 days (yes/no).

Our primary and most of our secondary outcomes are self-reported. Self-report measures of drug-related risk behaviours are deemed reliable, particularly when used with computer assisted survey instruments [43, 44]. The use of self-reported measures in evaluating the impact of harm reduction interventions such as NSPs is widespread [45]. Self-reported crack pipe sharing has been used as an outcome measure for Canadian studies [18, 27, 46] aiding comparability. Service presentations will be assessed through the collection of minimum identifiers (initial and date of birth) for participants in pre- and post-surveys and from each person obtaining a SIPP kit as part intervention monitoring for the process evaluation (see below). These minimum identifiers will be used to link data from surveys and SIPP monitoring, and also allow linkage of both survey and SIPP monitoring data with specialist drug services client lists before and after the intervention to review increased service presentation due to SIPP.

##### Recruitment

We will recruit up to 740 people who use crack through participating specialist drug services at the intervention (*n* = 400) and control sites (*n* = 200) and via peer networks (*n* = 140). Peer network recruitment will happen at both intervention (*n* = 100) and control sites (*n* = 40).

We will recruit using both randomised and targeted sampling. We will attempt to recruit a randomised sample from specialist drug services, to reduce selection bias, but will be pragmatic considering difficulties in recruiting this marginalised population. The LSHTM team will work with specialist drug services to generate a randomised anonymised sample of clients who are eligible to participate in the study. The specialist drug services will start recruitment from this list contacting potential clients in line with standard service engagement practice and where appropriate invite people in to participate in the research. Should we fail to meet recruitment targets at week eight, recruitment will be opened to all eligible clients, irrespective of the list, including those attending for appointments, opportunistic walk-ins and referrals from friends. In addition, we will employ targeted sampling methods to recruit 140 participants representing diverse crack user sub-populations via peer networks to widen the evidence base on health needs and service access among people who use crack not currently engaging with services. Both pre- and post-surveys recruitment will be conducted over the course of 10 weeks with an additional 4-week extension if necessary.

##### Sample size

**Fig. 3:**
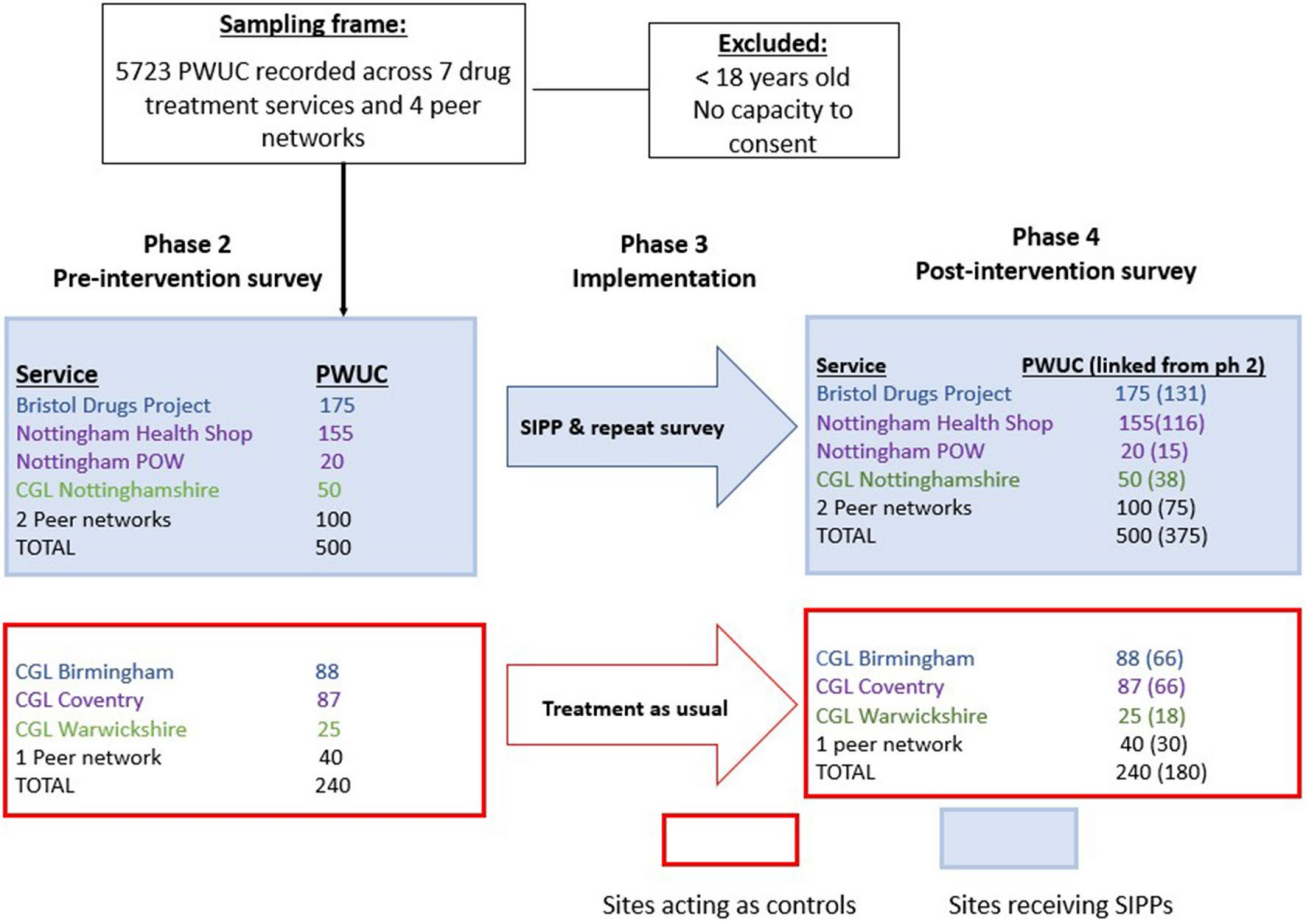
Impact evaluation sampling strategy

A sample of 306 (204 SIPP, 102 control) is sufficient to compare proportional differences in pipe sharing at 90% power with significance of *p* = 0.05. We assume 52% will share pipes, and that there will be a difference of 20% (52% to 32%) between control and intervention; substantiating previous evidence suggesting pipe provision reduces prevalence of sharing from 37 to 12% over a 6 month period [16]. An inflated sample of 740 (500 SIPP, 240 control) allows for linkage of 75% of participants between baseline and follow-up surveys and for multivariable analyses to adjust for any baseline differences in characteristics between intervention and control sites (see Fig. 3).

##### Analysis

We will analyse quantitative data in four ways. Firstly, we will describe demographic characteristics, drug use behaviours, and indicators of social exclusion among PWUC stratified by exposure group at the pre- and post-survey time points. Secondly, we will estimate the change in primary outcome (sharing of pipes in past 28 days) for participants in intervention sites compared to control sites. Logistic regression will be used to compare baseline prevalence of primary and secondary outcome measures between intervention and control sites post-intervention. Primary analysis will use an adjusted, individual-level intention-to-treat approach, including all participant data (irrespective of baseline and follow-up participation, their engagement with SIPP and assuming missing observations are missing at random) in order to reflect the effects of SIPP in every day practice. The same analysis plan will be used for secondary outcomes (use of homemade pipes, cuts/burns, injecting, and service engagement). Linear regression models will compare baseline frequency of injecting between intervention and control sites and estimate relative changes in mean frequency of injecting. Logistic and linear models will adjust for a priori confounders including sex, age, duration of crack use, engagement in services, primary outcome measures, and any variables with evidence of imbalance described above. As a sensitivity analysis will focus on participants for which we have baseline and follow-up (though data linkage) to assess changes in outcomes over time and between intervention and control. Thirdly, we will undertake a per-protocol analysis, limiting the analyses to participants with both baseline and follow-up data and who report use of SIPP. This will give a measure of efficacy where SIPP is delivered in the optimal way.

Appropriate statistical models will be selected based on evidence of clustering observed. The model will include fixed effects for time and treatment, and we will explore the appropriateness of including a random effect to account for heterogeneity of participants within sites or a fixed effect to account for heterogeneity across sites. We will explore the need to adjust for clustering within treatment site and by sites using intra-class correlation coefficients. Fourthly, we will examine evidence of a dose–response relationship between intensity of exposure to SIPP and primary and secondary outcomes using logistic regression models and categorising exposure to SIPP as a continuous or categorical variable (1, 2–3, 4–5,6 + exposures) depending on the distribution of contacts with SIPP in the intervention sites.

#### 3. Process evaluation

Our process evaluation is informed by realist approaches to intervention theory [47] which stress the importance of context in understanding mechanisms of change; what “works” in one time and place may be ineffective, or even harmful, elsewhere. This is reflected in our working process evaluation framework, which guides exploration of key process evaluation domains as detailed in Table 3.

**Table 3:** Working process evaluation framework

Research domain		Research questions
Implementation	Fidelity & quality	How did implementation of SIPP intervention vary from what was planned, i.e. (a) recruitment of PWUC at all sites, (b) training and protocols delivered, (c) provision of SIPP kits, (d) active engagement of peer networks, (e) SIPP harm reduction materials developed and used
*What is implemented and how?*		What were the barriers and facilitators to implementation fidelity?
		What adaptations were made?
	Coverage (reach & dose)	How many: (a) PWUC were approached across all sites, (b) PWUC took up SIPP kits and/or peer-led harm reduction training (c) PWUC were referred or linked to specialist drug services, and (d) local law enforcement and/or government authorities maintained support
		What were the barriers and facilitators to each of the above?
Mechanism of impact	Acceptability & feasibility	Which components of the intervention were best accepted and adopted by PWUC, peer-network members, providers, and health system/policy stakeholders? What were the experiences and perceptions of PWUC who were actively, somewhat or not at all engaged with harm reduction and SIPP uptake? What were the challenges and barriers faced?
*How does intervention lead to change?*	Interactions & consequences	How did various components of the intervention interact (i.e. SIPP kit provision, peer-network harm reduction outreach, uptake of specialist drug services)?
		Were there any unanticipated pathways or consequences?
Context	Proximal and distal	What social, cultural, political, and logistical factors impede or facilitate (or were affected by) how the intervention was implemented across the different sites
*How context affects implementation & outcomes?”*		What were contextual reasons for adaptations to the intervention and its delivery?

##### Sample

We will interview 36–45 people who use crack across the intervention sites (12–15 per site), with interviews spanning the pre-post-survey and intervention periods. We will purposively sample for variation in gender, ethnicity and age. A smaller number of people (6–9), sampled for duration of crack use (5 + years), will be recruited at the control sites (2–3 per site) to obtain an understanding of local crack use practices and market dynamics. Peers and providers involved in delivering the SIPP survey and intervention will facilitate recruitment. Clients meeting our purposive sampling criteria will be informed of the qualitative arm of the study and provided with a participant information sheet. If interested, they or the peer/provider can facilitate research team contact. We will also interview 15–18 service providers and peer researchers involved in survey and intervention delivery across the sites and select stakeholders (police, commissioners) purposively sampled to reflect variation in relevant occupational roles (*n* = 5–8). Stakeholders will be recruited through professional networks, and peers/providers through the engagement of the research team at each site. Total qualitative interviews 62–80. Flexibility in recruitment numbers allow for theoretical sampling. This is a grounded theory technique whereby early analysis of purposively sampled participant data allows for additional targeted sampling to address analytic gaps, puzzles or leads [48].

##### Data generation

We will employ qualitative methods (ethnographic observations, in-depth interviews) to: (i) explore the local social contexts and relations of drug use, risk practices, health concerns and service engagement/need over time (from pre to post-intervention), (ii) document and assess delivery of each component of the SIPP intervention, (iii) explore client and provider expectations, experiences, perceptions and interactions with the SIPP intervention; (iv) examine local contextual factors that may influence the effectiveness of the intervention as designed. Ethnographic observations will be recorded in anonymised field notes by each research team member, detailing each study site visit from pre through to post-intervention. Together, these will elaborate the local contextual features of each site, with attention to the social relations, resources and environments informing practices of drug use, risk and care, as well as the internal/external relations and resources informing drug service and policing practice and policy. Interviews will be informed by topic guides oriented to exploring the domains noted above (i to iv). Finally, monitoring data collected by service providers alongside SIPP kit delivery will enable assessment of intervention coverage and reach (e.g. how many SIPP kits were distributed and to whom). Monitoring data categories comprise: place of engagement; numbers of pipes received; reason for new SIPP kit; component of kit received (whole kit, mouthpiece, filter, push stick); gender; ethnicity; engagement with service; reason for engagement; minimal identifiers (date of birth, initials).

##### Analysis

Interviews will be recorded and transcribed verbatim. Observation fieldnotes will be generated throughout and integrated alongside transcripts in analyses. Qualitative data will be managed in NVivo Software. We will conduct a thematic analysis comprising six stages: (i) data familiarisation; (ii) first-level open coding, (ii) coding framework development; (iii) second level inductive coding; (iv) category mapping; (v) thematisation; and (vi) write up. This process is a modification of Braun and Clarke’s [49] guidelines for thematic analysis, also incorporating grounded theory principles such as early analysis alongside data generation, process-orientated inductive coding and theoretical sampling [48].

We will triangulate analysis [50] with attention to: (a) multiple forms of qualitative data (interviews, observations); (b) multiple participant perspectives (service providers, stakeholders, PWUC from diverse communities, treatment engaged/disengaged); (c) multiple intervention sites; and (d) multiple time points (pre/during/post-intervention). The primary focus of triangulation will be to identify congruence and divergence, as well as to maximise the confidence with which judgements are made regarding potential relative intervention effects. Where possible, quantitative and qualitative analyses will build upon one another, qualitative to explore baseline survey data findings and to inform the impact evaluation – by providing insight into local secular changes at intervention sites, not captured by comparison site data. We will hold data analysis workshops with peer network and advisory board members, to "sense check" our interpretations, co-produce visual category mapping and discuss community feedback mechanisms, including through presentations and resource development.

To measure coverage including reach and dose of SIPP we will analyse monitoring data collected alongside the SIPP Kit delivery (see data generation above). Firstly, minimum identifiers will be used to link those that received SIPP with those that took a baseline and follow-up survey to assess what proportion of the population engaged with SIPP. Secondly, together with information on intervention activities (e.g. number of SIPP kits distributed), monitoring data will be analysed descriptively to assess the fidelity of the intervention (the extent to which it is delivering what it set out to do); intensity in which SIPP kits are used and by which population (age, gender, ethnicity); and reach (what proportion of the population are engaging in SIPP at each site). This will include assessing the lifespan of a SIPP kit, the number of first-time recipient's vs re-occurring recipients and trends in the number of SIPP kits being distributed during the intervention.

#### 4. Economic evaluation

Economic evaluation of SIPP will consist of estimation of the costs and cost-effectiveness of the intervention. Prior to starting cost data collection, we will undertake a scoping review of the literature to identify evidence that links reported changes in behaviour, specialist drug or health service engagement, or safe drug taking practices to longer term outcomes, including blood-borne virus transmission, respiratory illness, mental health, drug-related deaths and criminal justice involvement. We will also host discussions with service providers, project team members, and commissioners to identify relevant outcomes for the decision-making context.

We will estimate the incremental, economic costs of the SIPP kits from the provider perspective following economic best practice [51]. Primary cost data will be collected from study sites and peer providers using an ingredients approach, where the value of inputs is based on quantities and unit prices, including staff salaries, building space, training, supplies, equipment and overheads. Our cost-effectiveness analysis will estimate the likelihood that the intervention is cost-effective as implemented in study sites, using estimates of treatment effects from the impact evaluation. We will explore potential future changes in key drivers of costs (including the costs of pipes and mode of delivery) in sensitivity analysis.

##### Impact and dissemination

Should SIPP be proven to be effective and cost-effective, we will work with the control and intervention sites to support continuation of police permissions for crack pipe supply, while advocating for inclusion of safe crack inhalation equipment as an exemption under Sect. 9A of the Misuse of Drugs Act 1971. There is appetite for this reform. Since study commencement, the team have fielded enquiries from drug treatment providers and policy makers across the UK about the study process, with interest in obtaining police approvals for local supply and the generation of evidence for broader reform. We have supported this process for sites that do not compromise our study integrity (i.e. not proximal to intervention or control sites) and are aware of one service that is now providing pipes with police approvals, with another in progress.

We will consolidate findings and work with community partners to develop an optimised SIPP kit and provider training package to inform best practice at any sites able to continue provision with local police permissions. Through widespread dissemination of findings through commissioner, policy and provider networks we will assess and catalyse the support required for implementation at scale. We have collated crack harm reduction resources available globally, reviewing these for their applicability to the UK context. These, and relevant qualitative data, will inform workshops with community members to co-develop harm reduction outputs for people who use crack. As part of this process, and drawing on learning from peer-network engagement, we will facilitate development of a tool kit to support community mobilisation, peer-led harm reduction and equipment supply.

## Discussion

In this paper we introduce the protocol for the NIHR-funded SIPP (Safe Inhalation Pipe Provision) Study; a novel intervention in the UK context. Although injecting equipment, such as syringes, filters, cookers and acidifiers, are freely available to people who use drugs, commensurate provision of equipment for crack smoking is prohibited under UK law. Under Sect. 9A of the Misuse of Drugs Act any supply of articles for the purposes of administering a controlled drug have to be listed as exempt from prohibition. Most injecting equipment (although not tourniquets) are listed as exempt, foil for heroin smoking was included in 2014, but no exemption is provided for inhalation equipment provision to people for the purposes of crack use. This lacuna in the law exacerbates health harms among an already highly marginalised and disenfranchised population. Our formative work to inform this proposal [12], supported by data generated since study commencement [52], show that use of unsafe homemade pipes for crack smoking is common across England. Restrictions on pipe provision are evidenced to increase pipe sharing, with associated viral transmission health impacts [18, 27–29].

The UK is a signatory to the 2016 Global Health Sector Strategy on viral hepatitis, with the 2030 goal of reducing hepatitis infections by 90% and deaths by 65% [53], yet new and repeat hepatitis C (HCV) infections remain high [19]. Provision of materials to support transitions from injecting (crack pipes, foil for heroin smoking [54]) are crucial to reduce injecting-related health harms, such as HCV. Here, our study has precedent. Until 2014 foil provision for smoking heroin was also omitted as an exemption under Sect. 9A of the Misuse of Drugs Act, and therefore prohibited. A small pilot evaluation study operating with police permissions in 2006–2007 found that foil provision supported transitions from heroin injection to smoking [54]. This evidence informed a legislative amendment in 2014 to allow for foil provision through drug treatment services and NSP [55–57]. Provision of foil for heroin smoking is now widespread and well accepted in the UK, but high levels of societal concern about crack means that additional evidence is required to demonstrate that these benefits are transferable across drugs.

The process by which SIPP could inform a review of and change to current UK legislation would involve working with public health leads, civil servants at the Department of Health and the Office of Health Inequalities and Disparities (OHID), and Parliamentarians who would champion the calls for a legislative review. The aim is to convince the Home Secretary to ask the Advisory Council on the Misuse of Drugs (ACMD) to carry out a review of the evidence for crack pipe provision. The ACMD is a statutory body established in accordance with Section 1 of the Misuse of Drugs Act 1971 to advise the Government on drugs. Although the ACMD’s advice is not binding on the Government it can be persuasive, including in the case of foil for heroin smoking [57].

Existing networks of supportive MPs and Peers are crucial to this process, and a number of All-Party Parliamentary Groups (APPGs) would be engaged to help persuade the Home Secretary to call on the ACMD to undertake a review of the evidence. The findings of this study will be key to informing any review. Beyond parliamentary engagement it will also be important to work with civil servants, locally and nationally, to support the call for review and a change to the legislation. Local public health commissioners will be asked to endorse the expansion of paraphernalia laws to allow for the supply of crack pipe as an effective tool to engage with, and reduce the health harms of, this community. Civil servants at the Department of Health and OHID will also be engaged to understand what the political barriers to reform might be allowing us to strategise on effective arguments for change.

Specialist services for people who use drugs in the UK currently offer a wide range of services but have little to offer PWUC. This is crucial to address, given that drug treatment service engagement is associated with reductions in morbidity, criminal justice involvement and drug-related deaths [58, 59]. The 2021 Carol Black report on drug use stressed the importance of reinvigorating UK service provision to reach “very vulnerable groups, such as crack cocaine users … [who] do not receive adequate or any service but are at great risk” [60]. The SIPP intervention offers potential for a pragmatic and meaningful point of contact with a high-risk population. Crucially, this is not just a pipe provision intervention but comprises comprehensive crack harm reduction training for providers, including in relation to respiratory health. Smoking risk is rarely prioritised in harm reduction interventions, which predominantly orientate around injecting practices, and provider confidence in engaging with people who use crack or assessing respiratory health can be low. The COVID-19 pandemic generated support for the SIPP project, given that pipe sharing poses a high COVID-19 transmission risk [61]. Smoking crack is associated with pulmonary and respiratory complications such as pulmonary oedema and COPD [23, 25]. Use of homemade pipes can exacerbate respiratory conditions – placing people at heightened risk of COVID-19 related morbidity if they contract the virus [12]. High prevalence of COPD among this population [35, 62] is a continuing concern even as COVID-19 risk diminishes with vaccine rollout.

## Conclusion

This project is a community-academic partnership, addressing community-identified unmet need and building peer researcher capacity, with meaningful peer and community stakeholder involvement throughout, ensuring project accountability and output relevance. Specialist services for people who use drugs currently have little to offer people who use crack, and crack harm reduction awareness among providers is generally low. Pipes, a highly valued commodity among people who smoke crack, can provide an engagement hook to assess unmet need, including in relation to respiratory health. Our research is imperative to innovate service provision to increase its relevance to people who use crack cocaine, to inform legislative review and reduce crack-related, injecting and respiratory harms among this highly disenfranchised and growing population.

## Data Availability

Not applicable. For additional study information see: https://www.lshtm.ac.uk/research/centres-projects-groups/sipp

## Abbreviations

COPD: Chronic obstructive pulmonary disease
COVID-19: Coronavirus disease
HCV: Hepatitis C virus
HIV: Human immunodeficiency virus
LSHTM: London School of Hygiene & Tropical Medicine
NIHR: National Institute for Health and Care Research
NSP: Needle and syringe programmes
ODK: Open Data Kit
OST: Opioid substitution therapy
PWUC: People who use crack
SIPP: Safe inhalation pipe provision
UK: United Kingdom
